# Pretreatment Neutrophil-to-Lymphocyte Ratio (NLR) May Predict the Outcomes of Advanced Non-small-cell Lung Cancer (NSCLC) Patients Treated With Immune Checkpoint Inhibitors (ICIs)

**DOI:** 10.3389/fonc.2020.00654

**Published:** 2020-06-23

**Authors:** Ye Li, Zhibo Zhang, Yi Hu, Xiang Yan, Qi Song, Guoqiang Wang, Runzhe Chen, Shunchang Jiao, Jinliang Wang

**Affiliations:** ^1^Department of Oncology, The First Medical Center of Chinese PLA General Hospital, Beijing, China; ^2^Oncology Laboratory, The First Medical Center of Chinese PLA General Hospital, Beijing, China; ^3^Medical School of Chinese PLA, Beijing, China; ^4^The 78th Group Army Hospital of Chinese PLA, Mudanjiang, China; ^5^The Medical Department, 3D Medicines Inc, Shanghai, China; ^6^Departments of Thoracic/Head and Neck Medical Oncology and Genomic Medicine, The University of Texas MD Anderson Cancer Center, Houston, TX, United States

**Keywords:** non-small-cell lung cancer, neutrophil-to-lymphocyte ratio, peripheral blood biomarker, immunotherapy, immune checkpoint inhibitors, prognosis

## Abstract

**Background:** Recent studies have demonstrated the predictive value of pretreatment neutrophil-to-lymphocyte ratio (NLR) in advanced cancers; however, the role of NLR in patients with advanced non-small-cell lung cancer (NSCLC) treated with immune checkpoint inhibitors (ICIs) remained to be explored. Thus, we aimed to investigate whether pretreatment NLR was associated with the outcomes of advanced NSCLC patients treated with ICIs.

**Methods:** A comprehensive literature research was first conducted in PubMed, the Cochrane Central Library, and Embase for studies that evaluated the association between pretreatment NLR and survival of advanced NSCLC patients with ICIs treatment. We then conducted a retrospective study in Chinese People's Liberation Army (PLA) General Hospital (Beijing, China) to validate these findings.

**Results:** A total of 17 eligible studies with 2,106 patients were included in our meta-analysis, of which, 12 studies reported progression-free survival (PFS), and 13 studies reported overall survival (OS). The pooled results showed that high pretreatment NLR was significantly associated with poorer PFS (HR = 1.44, 95% CI 1.26–1.65; *P* < 0.001) and OS (HR = 2.86, 95% CI 2.11–3.87; *P* < 0.001) compared with those with low pretreatment NLR. Subgroup analysis demonstrated that the association between baseline NLR and PFS remained significant except that the cut-off value of NLR was 3 (HR = 1.48, 95% CI 0.93–2.37; *P* = 0.098) and region of Asia (HR = 1.55, 95% CI 1.00–2.39; *P* = 0.051). These results were further validated in our retrospective study that patients with pretreatment NLR ≥ 6.0 had shorter PFS (median: 5.0 vs. 9.1 months, HR = 1.39; 95% CI 1.01–1.91; *P* = 0.02) and OS (median: 10.0 vs. 17.3 months, HR = 1.71; 95% CI 1.18–2.46; *P* < 0.001) compared with those with NLR < 6.0. The associations between NLR and survival were consistent in subgroup analysis stratified by age, gender, ECOG PS, histology, stage, smoking history, treatment, and prior lines of therapy. Dynamics of NLR (dNLR) that increased ≥3.0 from baseline was also significantly associated with worse PFS (median: 3.1 vs. 9.1 months; *P* = 0.01) and OS (median: 6.8 vs. 17.0 months; *P* < 0.0001).

**Conclusions:** Our study demonstrates that pretreatment NLR and dNLR from baseline are associated with the outcomes of advanced NSCLC patients treated with ICIs; however, it warrants further prospective studies.

## Introduction

Primary lung cancer is one of the most common malignant neoplastic diseases. Non-small-cell lung cancer (NSCLC) accounts for about 80% of primary lung cancer, mainly consisting of adenocarcinoma and squamous cell carcinoma (1). Although the survival time for patients with lung cancer has been improved over the past few decades, disease prognosis and treatment outcomes are not satisfactory. In addition, the 5-year survival rate of lung cancer remains low worldwide (2, 3). Thus, effective therapeutics are still in urgent demand.

With the increasing awareness of the role of the immune system in tumor development and response, immunotherapy has received increasing attention and plays a crucial role in current cancer treatment (4, 5). In particular, the emergence of immune checkpoint inhibitors (ICIs) has led to a paradigm shift in the field of NSCLC treatment (6). However, not all patients are responsive to ICI therapy. Certain biomarkers, such as programmed cell death-ligand 1 (PD-L1), tumor mutational burden (TMB), and neoantigen load, which may reflect the state of tumor immune microenvironment, have shown utilities in selecting patients who are likely to benefit from ICIs treatment. However, the detection of these biomarkers depends greatly on the adequacy of tumor tissue. Thus, biomarkers that can be conveniently evaluated in a non-invasive manner are urgently needed.

Previous studies have shown that tumor-related inflammation is associated with the prognosis of solid tumors (7–14), which is not only crucial in different stages of cancer development, including initiation, promotion, invasion, and distant metastasis (15, 16) but also can affect the host's immune response to cancer (16–18). In the clinic, hematological indicators are commonly adopted to assess systemic inflammation, including white blood cells and C-reactive protein (CRP). In addition, the neutrophil-to-lymphocyte ratio (NLR) has become a recognized indicator of systemic inflammation (19, 20). Recently, studies have demonstrated the predictive value of pretreatment NLR in advanced cancers, including gastric cancer, liver cancer, and breast cancer (21–24). However, studies on the relationship between NLR and the prognosis of NSCLC are still limited, and the results appear inconsistent (25, 26).

Thus, our study was aimed to study the association between pretreatment NLR and survival in patients with NSCLC treated with ICIs. We first reviewed the literature to pool analyze the association between pretreatment NLR and clinical outcomes of advanced NSCLC patients receiving immunotherapy, and then, we conducted a retrospective study to validate these results.

## Materials and Methods

### Systematic Literature Review

#### Search Strategy and Study Selection

We performed a comprehensive online search using PubMed, Embase, and Cochrane Library (update on February 29, 2020). The terms used for online searching included “non-small-cell lung cancer,” “NSCLC,” “lung cancer,” “neutrophil-to-lymphocyte ratio,” “NLR,” “immunotherapy,” “immune checkpoint inhibitor,” “ICI,” “programmed death-1 receptor,” “PD-1 inhibitor,” “programmed death ligand-1,” “PD-L1 inhibitor,” “cytotoxic T lymphocyte antigen-4,” “CTLA-4,” “pembrolizumab,” “nivolumab,” “ipilimumab,” “avelumab,” “atezolizumab,” “durvalumab,” “predict,” “predictive,” “predictor,” “prognostic,” and “prognosis” (27). Both medical subheadings (Mesh) terms and free text were used in the search strategy. The retrieval formula is shown in the supplements.

Studies eligible for inclusion met the following criteria: (1) studies on immunotherapy for advanced NSCLC patients; (2) analysis of the association between prognosis and pretreatment NLR; (3) hazard's ratio (HR) with 95% CI was provided for PFS and/or OS according to NLR; (4) the full text was obtained.

Exclusion criteria were as follows: (1) duplicated study; (2) combination use of chemotherapeutics and ICIs; (3) insufficient usable data; (4) reviews, case reports, or unrelated articles.

#### Data Extraction

The following data were extracted from eligible studies: name of the first author, study design, published year, region of study, the total number of patients, gender, age, pathology, type of ICIs, cut-off value of NLR, follow-up period, and outcome of interest. Extraction of HRs and the related 95% CIs for PFS or OS was performed independently by two investigators (Ye Li and Zhibo Zhang). Any discrepancy was solved in discussion. The review was based on the Preferred Reporting Items for Systematic Reviews and Meta-Analyses (PRISMA).

#### Quality Assessment

As mentioned in the previous study (28), two investigators (Ye Li and Zhibo Zhang), respectively, evaluated the quality of the eligible studies using the Newcastle–Ottawa Scale (NOS), which has been validated for evaluating the quality of studies (29). A “star-system” in NOS form was used to assess study quality with scores ranging from 0 to 9 stars. A score >7 indicated a high quality given that grading criteria have not been defined.

### Retrospective Study

#### Study Population

We further aimed to determine whether baseline NLR and the change in NLR after 6 weeks from baseline were associated with outcomes in Chinese patients with advanced NSCLC receiving ICI therapy at the Chinese People's Liberation Army (PLA) General Hospital. Patients with advanced NSCLC receiving ICI treatment were retrieved from January 2015 to January 2019. Inclusion criteria: (1) patients with histologically confirmed advanced NSCLC (stage IIIB–IV according to the eighth edition of TNM staging system for lung cancer) (30); (2) patients received ICI therapy. Exclusion criteria: (1) patients treated with ICIs less than two cycles; (2) patients without efficacy evaluation; (3) patients without blood routine examination at baseline and after two cycles of treatment. The retrospective study involving human participants was reviewed and approved by the Ethics Committee of Chinese PLA General Hospital.

#### Data Collection

We collected the clinical characteristics of the patients, blood routine test at baseline and 6 weeks after treatment, the efficacy evaluation of immunotherapy, as well as prognostic information. Clinical characteristics included age, gender, Eastern Cooperative Oncology Group Performance status (ECOG PS), histology, stage, smoking history, treatment (monotherapy/combination therapy), and prior lines of therapy. The value of NLR was calculated using the absolute value of neutrophils and lymphocytes.

Response Evaluation Criteria in Solid Tumors (RECIST) 1.1 was applied to evaluate the efficacy of treatment, including complete response (CR), partial response (PR), stable disease (SD), and progressive disease (PD). Patients with an efficacy evaluation of CR or PR were confirmed by imaging examination after 4 weeks. Progression-free survival (PFS) was determined from the date of the first ICI treatment to true progression or death due to any cause, or censored at the date of last patient contact; overall survival (OS) was determined between the date of ICI initiation treatment and death of any reason or the last date of patient contact (which occurs first). All patients were followed up by telephone counseling and medical records reviewing, and cut-off date was September 15, 2019.

### Statistical Analysis

We pooled the HRs with 95% CIs of PFS and OS, using the method of random-effects inverse-variance-weighted to estimate the size of the treatment benefit. *I*^2^ statistics were used to assess statistical heterogeneity and the extent of variability attributable to any heterogeneity across different studies. No significant heterogeneity was found between studies when *P* > 0.1 and *I*^2^ < 50%. If there was no significant heterogeneity, a fixed effects model was used to calculate the pooled effect; otherwise, a random effect model was used. Publication bias was assessed using *Begg's* and *Egger's tests*. All statistical analyses were performed using *STATA 15.1* and *SPSS 21.0*. The Kaplan-Meier method was used to assess survival, and survival curves were compared through the log-rank test with HR and 95% CI determined by Cox regression. The nominal level of significance was set at 5%, and all *P*-values were two sided.

## Results

### Search Strategy

**Figure 1:**
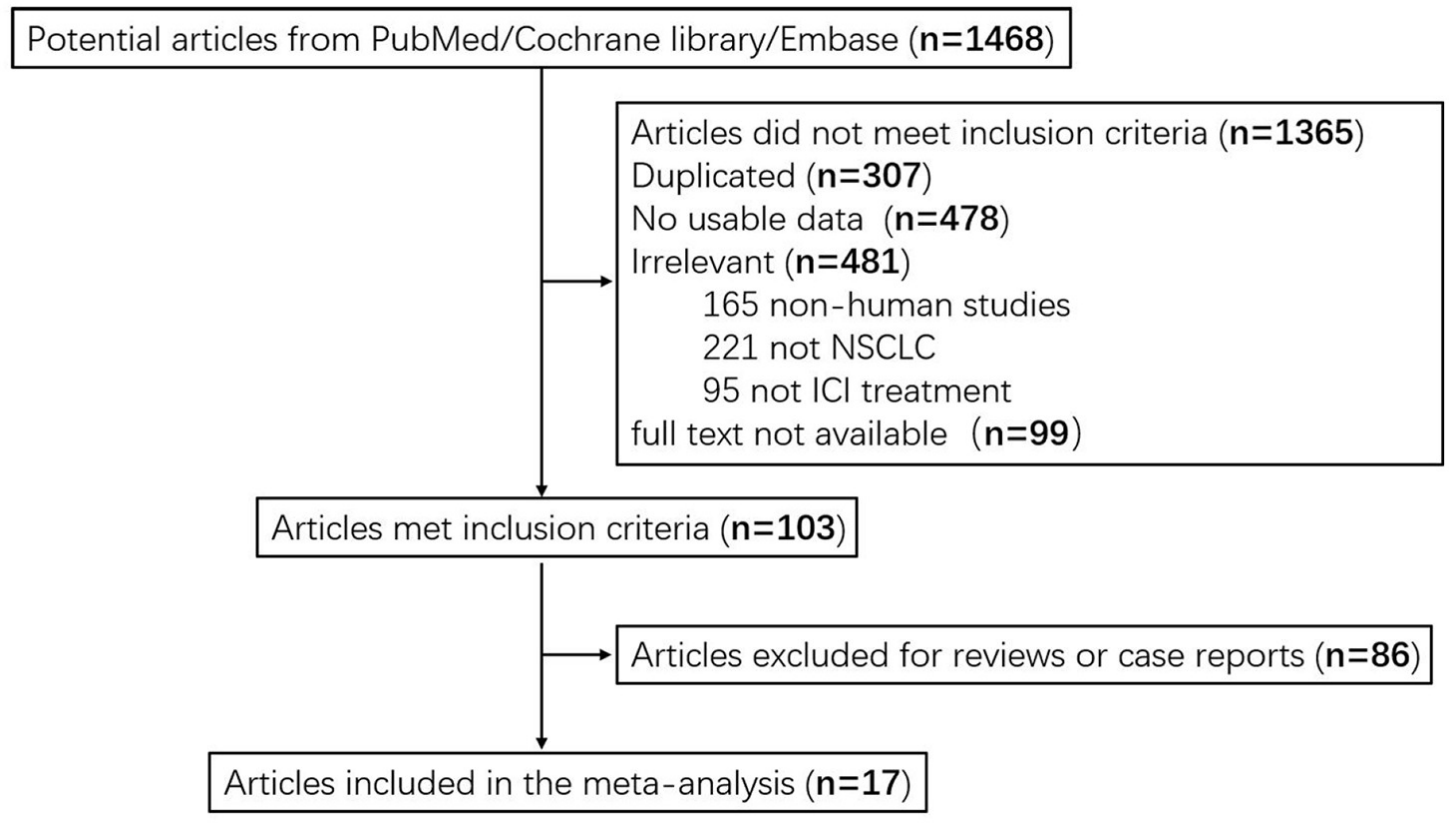
Flow chart of eligible studies in meta-analysis.

A total of 1,468 articles were identified in the systematic searching. After the review of the titles and abstracts, 1,365 articles failed to meet the inclusion criteria. In total, 307 articles were excluded due to duplicate records, 478 were excluded due to insufficient usable data, 481 were excluded due to low correlation, and 99 were excluded as lack of full text. After further reading the entire articles, we excluded 86 case reports or reviews. Seventeen studies were included in the pool analysis (7, 31–39). Data from eligible studies were acquired from published articles. A flow diagram describing selection of included studies is shown in Figure 1.

### Characteristics of Eligible Studies and Quality Assessment

Seventeen retrospective studies with 2,106 patients published from 2017 to 2020 were included in the pool analysis. We summarized the characteristics in Table 1. Of all included studies, five were performed in the United States (7, 31, 33, 34, 40), four in Japan (32, 36, 39, 44), and the remaining eight studies were conducted in Switzerland (35), Canada (37), France (38, 45), Bulgaria (41), China (42), Germany (43), and Italy (46), respectively. In terms of the ICIs used, 11 studies reported on nivolumab (7, 31–36, 39, 42, 45, 46), 2 studies reported on pembrolizumab (41, 44), and the remaining 4 studies did not specify the type of ICIs (37, 38, 40, 43). The most common cut-off value of NLR was 5. Twelve studies reported the association between pretreatment NLR and PFS for advanced NSCLC patients receiving immunotherapy, and 13 studies reported on OS. The quality results for all eligible studies assessed by Newcastle–Ottawa Scale (NOS) are listed in Table 2. Nine studies got quality scores of 7 stars and eight studies got 8 stars.

**Table 1:** Characteristics of eligible studies on prognostic utility of the NLR in NSCLC patients receiving ICI.

**References**	**Study design**	**Year**	**Region**	**Sample**	**Male/female**	**Age (year), Median (range)**	**Squamous carcinoma/ adenocarcinoma/others**	**ICI**	**Calculation**	**Cut-off value**	**Follow-up period (months)**	**Outcome**
Bagley et al. (7)	Retrospective cohort	2017	USA	175	80/95	68 (33–88)	42/133 (Squamous/non-squamous)	Nivo	Baseline	5	NR	PFS, OS
Park et al. (31)	Retrospective cohort	2017	USA	159	82/77	68 (41–91)	109/39/11	Nivo	Baseline	5	11.5 (9.4–13.1)	PFS
Kataoka et al. (32)	Retrospective cohort	2017	Japan	189	139/50	69 (38–88)	NR	Nivo	Baseline	5	5.5	PFS
Rogado et al. (33)	Retrospective cohort	2017	USA	40	29/11	67	NR	Nivo	Baseline	5	NR	PFS, OS
Patil et al. (34)	Retrospective cohort	2017	USA	115	48/67	67	NR	Nivo	Baseline	2.8	NR	PFS
Diem et al. (35)	Retrospective cohort	2017	Switzerland	52	29/23	68	18/30/4	Nivo	Baseline	5	14.0	PFS, OS
Shiroyama et al. (36)	Retrospective cohort	2018	Japan	201	135/66	68 (27–87)	41/160 (Squamous/non-squamous)	Nivo	Baseline	4	12.4	PFS
Zer et al. (37)	Retrospective cohort	2018	Canada	88	43/45	64 (31–81)	15/66/7	PD-1/PD-L1	Baseline	4	5.3 (0.4–38.1)	PFS, OS
Mezquita et al. (38)	Retrospective cohort	2018	France	466	301/165	62 (29–86)	159/270/37	PD-1/PD-L1	Baseline	3	12.0 (11.0–14.0)	PFS, OS
Fukui et al. (39)	Retrospective cohort	2018	Japan	52	37/15	69 (46–83)	16/33/3	Nivo	Baseline	5	10.9 (5.6–16.4)	OS
Maymani et al. (40)	Retrospective cohort	2018	USA	74	36/38	68	12/62 (Squamous/non-squamous)	Nivo/Pembro/Durva	Baseline	6	12.3	OS
Petrova et al. (41)	Retrospective cohort	2019	Bulgaria	119	74/45	62	51/68 (Squamous/non-squamous)	Pembro	Baseline	5	——	PFS, OS
Liu et al. (42)	Retrospective cohort	2019	China	44	33/11	60 (43–74)	13/31/0	Nivo	Baseline	3.07	6.9 (0.6–28.5)	PFS, OS
Möller et al. (43)	Retrospective cohort	2019	Germany	35	19/16	65 (24–85)	7/23/5	Nivo/Pembro	Baseline	5.2	9.7 (1.0–26.0)	OS
Hasegawa et al. (44)	Retrospective cohort	2019	Japan	51	40/11	70 (35–86)	16/35 (Squamous/non-squamous carcinoma)	Pembro	Baseline	4.56	9.5 (0.5–25.6)	PFS, OS
Dusselier et al. (45)	Retrospective cohort	2019	France	59	44/15	60 (30–87)	12/47 (Squamous/non-squamous carcinoma)	Nivo	Baseline	5	——	OS
Russo et al. (46)	Retrospective cohort	2020	Italy	187	137/50	67 (34–83)	86/101 (Squamous/non-squamous carcinoma)	Nivo	Baseline	5	——	OS

*NR, not reported; HR, hazard ratio; OS, overall survival; PFS, progression-free survival; NLR, neutrophil-to-lymphocyte ratio; ICI, immune checkpoint inhibitor; Nivo, Nivolumab; Pembro, pembrolizumab; Durva; durvalumab; PD-1, programmed cell death-1; PD-L1, programmed cell death-ligand 1*.

**Table 2:** Methodological characteristics of eligible studies and quality score.

**References**	**Representativeness of population**	**Sample size**	**Non-respondents**	**Ascertainment of the exposure (risk factor)**	**Comparability**	**Assessment of the outcome**	**Statistical test**	**Total stars**
Bagley et al. (7)	*	*	*	**	*	*	*	8
Park et al. (31)	*	*	*	**	*	*	*	8
Kataoka et al. (32)	*	*	*	**	*	*	*	8
Rogado et al. (33)	*	—	*	**	*	*	*	7
Patil et al. (34)	*	*	*	**	*	*	*	8
Diem et al. (35)	*	—	*	**	*	*	*	7
Shiroyama et al. (36)	*	*	*	**	*	*	*	8
Zer et al. (37)	*	—	*	**	*	*	*	7
Mezquita et al. (38)	*	*	*	**	*	*	*	8
Fukui et al. (39)	*	—	*	**	*	*	*	7
Maymani et al. (40)	*	—	*	**	*	*	*	7
Petrova et al. (41)	*	*	*	**	*	*	*	8
Liu et al. (42)	*	—	*	**	*	*	*	7
Möller et al. (43)	*	—	*	**	*	*	*	7
Hasegawa et al. (44)	*	—	*	**	*	*	*	7
Dusselier et al. (45)	*	—	*	**	*	*	*	7
Russo et al. (46)	*	*	*	**	*	*	*	8

One “*” means one point.

### Association Between Pretreatment NLR and PFS in Eligible Studies

**Figure 2:**
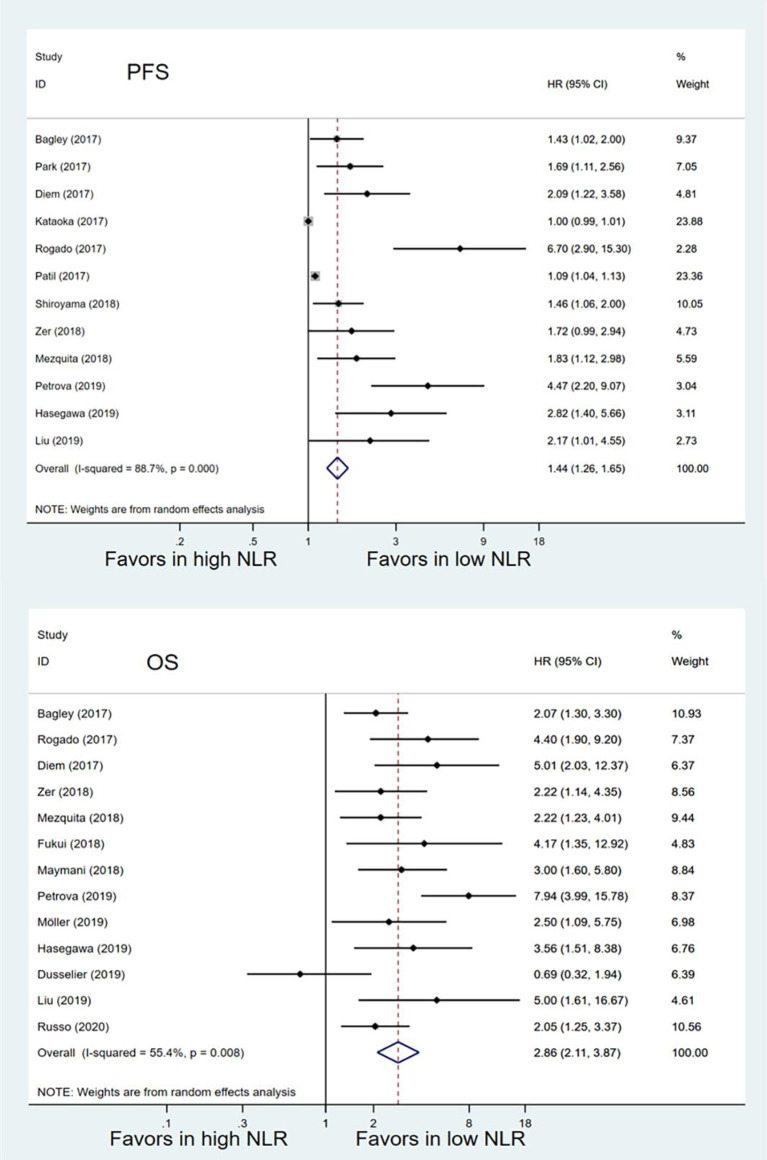
Meta-analysis of the associations between pretreatment neutrophil-to-lymphocyte ratio (NLR) and progression-free survival (PFS) or overall survival (OS).

Twelve studies with 1,699 patients were finally included to analyze the association between pretreatment NLR and PFS. The pooled result suggested that high pretreatment NLR was significantly associated with poorer PFS (HR = 1.44, 95% CI 1.26–1.65; *P* < 0.001) (Figure 2). Subgroup analysis demonstrated that the association between baseline NLR and PFS remained significant except for the cut-off value of NLR was 3 (HR = 1.48, 95% CI 0.93–2.37; *P* = 0.098) and region of Asia (HR = 1.55, 95% CI 1.00–2.39; *P* = 0.051) (Table 3).

**Table 3:** Subgroup analyses of the associations between NLR and survival.

**Subgroup**	**No. of studies**	**Test of association**			**Test of heterogeneity**	
		**HR**	**95% CI**	***P*-value**	***I^**2**^*(%)**	***P*-value**
**Progression-free survival**						
Total	12	1.44	1.26–1.65	<0.001	88.7	<0.001
**Publication year**						
2017	6	1.20	1.06–1.37	0.004	90.5	<0.001
2018	3	1.59	1.25–2.02	<0.001	0.0	0.714
2019	3	3.05	2.01–4.62	<0.001	0.0	0.377
**Region**						
Asia	4	1.55	1.00–2.39	0.051	83.3	<0.001
Europe and America	8	1.98	1.38–2.84	<0.001	86.3	<0.001
**Sample size**						
>100	7	1.22	1.09–1.38	0.001	88.9	<0.001
≤100	5	2.17	1.01–4.55	<0.001	48.9	0.098
**NLR cut-off**						
>3	3	1.48	0.93–2.37	0.098	73.1	0.024
>4	3	1.73	1.24–2.41	0.001	29.9	0.240
>5	6	2.09	1.28–3.40	0.003	90.9	<0.001
**Follow-up period (months)**						
>12	1	1.46	1.06–2.01	0.019	–	–
≤12	6	1.68	1.13–2.50	0.010	82.3	<0.001
NR	5	2.24	1.30–3.86	0.004	90.3	<0.001
**Quality score**						
7	5	2.54	1.68–3.83	<0.001	48.9	0.098
8	7	1.22	1.09–1.38	0.001	88.9	<0.001
**Overall survival**						
Total	13	2.86	2.11–3.87	<0.001	55.4	0.008
**Publication year**						
2017	3	3.25	1.77–5.97	<0.001	55.2	0.107
2018	4	2.57	1.82–3.64	<0.001	0.0	0.718
2019	5	3.02	1.30–7.01	0.001	78.7	0.001
2020	1	2.05	1.25–3.37	0.005	–	–
**Region**						
Asia	3	4.05	2.25–7.31	<0.001	0.0	0.898
Europe and America	10	2.67	1.88–3.79	<0.001	63.5	0.003
**Sample**						
>100	4	2.83	1.62–4.93	<0.001	75.4	0.007
≤100	9	2.90	1.98–4.24	<0.001	44.5	0.071
**NLR cut-off**						
>3	2	2.83	1.37–5.87	0.005	32.3	0.224
>4	2	2.66	1.57–4.50	<0.001	0.0	0.395
>5	9	2.90	1.91–4.39	<0.001	67.6	0.002
**Follow-up period (months)**						
>12	1	3.00	1.58–5.71	0.001	–	–
≤12	6	2.72	1.96–3.76	<0.001	0.0	0.728
NR	6	3.25	1.77–5.97	0.001	55.2	0.107
**Quality score**						
7	9	2.90	1.98–4.24	<0.001	44.5	0.071
8	4	2.83	1.62–4.93	<0.001	75.4	0.007

*NR, not reported; HR, hazard ratio; NLR, neutrophil-tof-lymphocyte ratio*.

### Association Between Pretreatment NLR and OS in Eligible Studies

Thirteen studies with 1,442 patients were included to analyze the relationship between pretreatment NLR and OS. Patients with high pretreatment NLR also had shorter OS (HR = 2.86, 95% CI 2.11–3.87; *P* < 0.001) (Figure 2) compared with those with low pretreatment NLR. Subgroup analyses also showed that the association between pretreatment NLR and OS was robust (Table 3). When stratified by the region, there was a marginal significance between high pretreatment NLR and worse OS in the region of Asia (HR = 4.05, 95% CI 2.25–7.31; *P* < 0.001) and the regions of Europe and America (HR = 2.67, 95% CI 1.88–3.79, *P* < 0.001). When stratified by cut-off value, study quality, and sample size, high pretreatment NLR remained significantly associated with inferior OS.

### Sensitive Analysis

**Figure 3:**
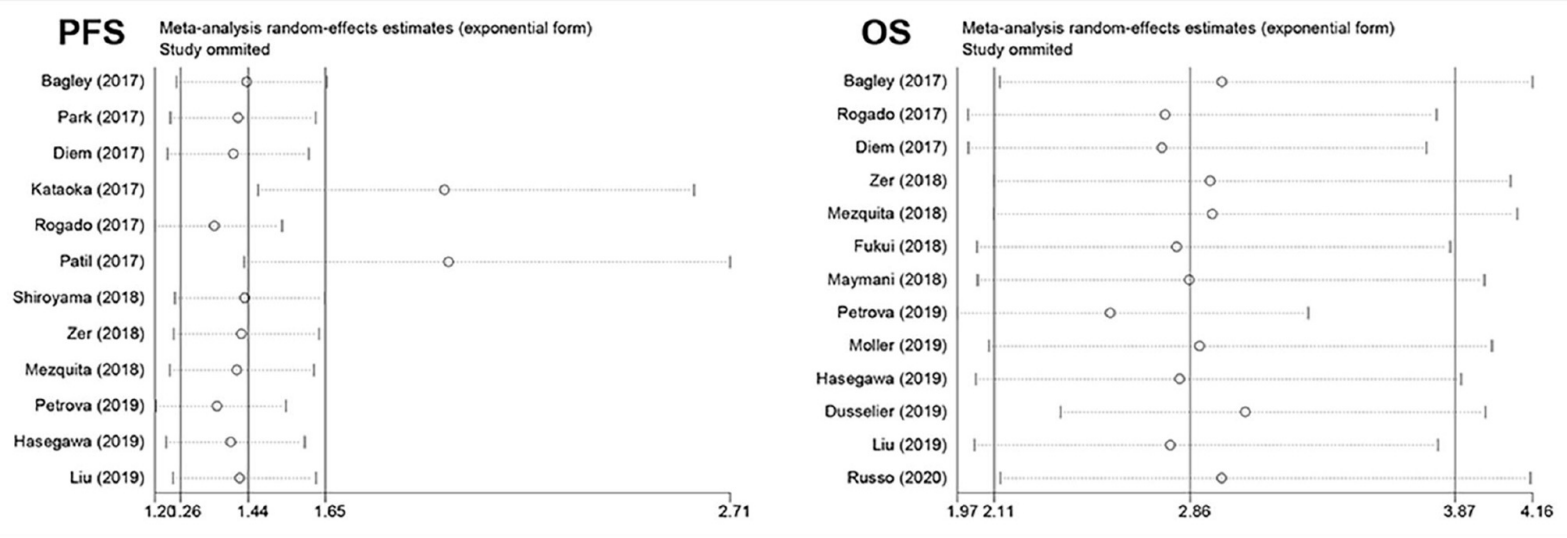
Plot of sensitivity analysis by excluding one study each time and the pooled estimates for the rest of the studies.

The pooled PFS showed that none of the individual studies have evident influence on the pooled result except for two studies conducted by Patil and Kataoka, which might affect the result, while the result was still significant. The pooled result for OS was still stable despite excluding each study separately, which suggested that the pooled result was robust (Figure 3).

### Publication Bias

The test results indicated no statistical publication bias in the HRs of PFS (*Begg's test, P* = 0.131; *Egger's test, P* = 0.073) or OS (*Begg's test, P* = 0.051; *Egger's test, P* = 0.271).

### Clinical Characteristics

**Figure 4:**
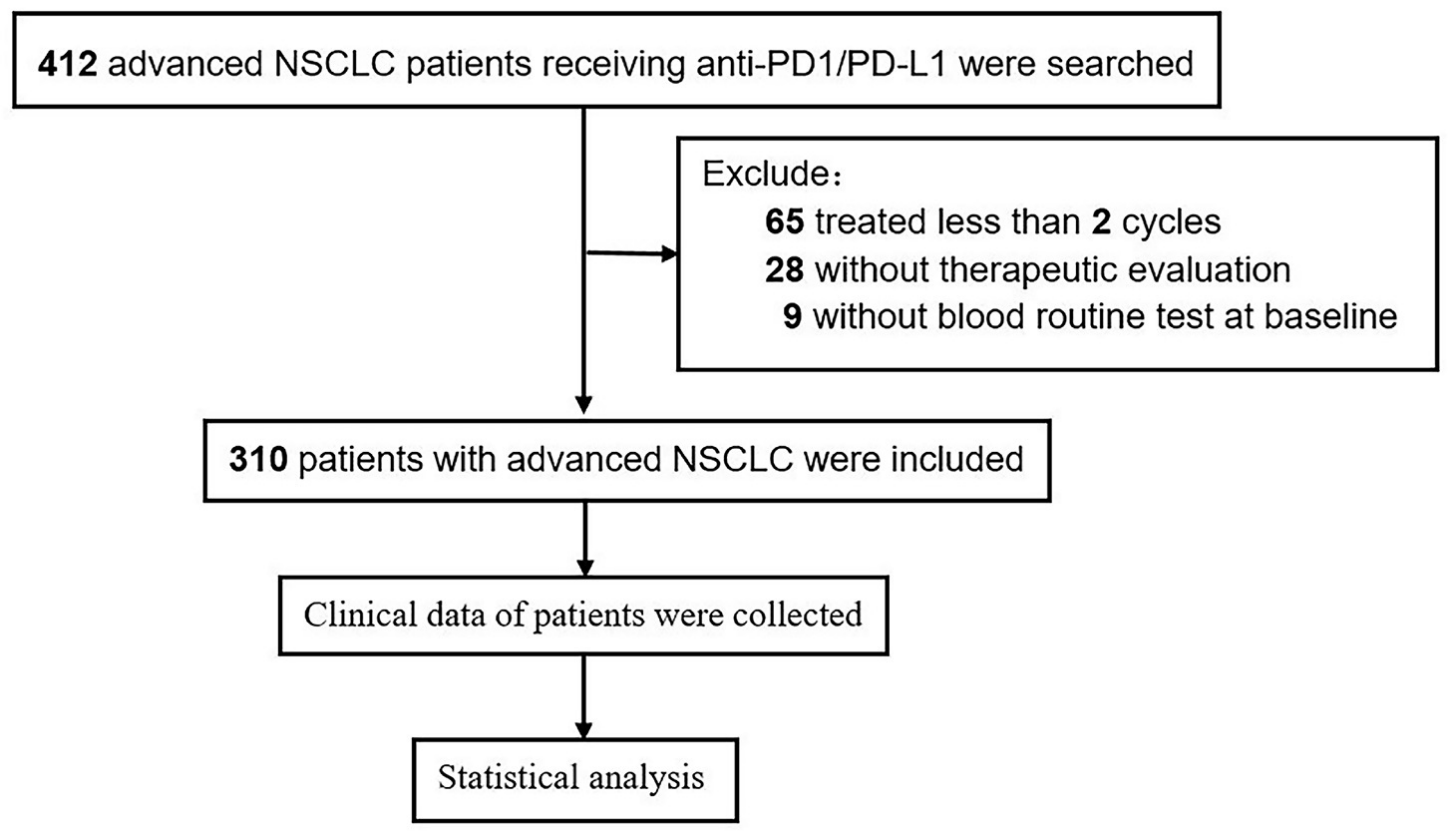
Flow chart of patients' selection in retrospective study.

A total of 310 patients with advanced NSCLC receiving ICI therapy were included in our study, of which 237 were males (76.5%). The median age was 61 years (range, 33–91). Patients (175; 56.5%) were with adenocarcinoma histology, 113 (36.5%) were with squamous cell carcinoma, and 22 (7.1%) were with other types. Patients (278; 89.7%) were with ECOG PS 0–1, and 193 (62.3%) were smokers. According to the International Lung Cancer Research Association eighth edition TNM staging, 66 patients (21.3%) were in stage IIIB/C, and 244 patients (78.7%) were in stage IV. Of the patients, 51.9% (*n* = 161) received combination therapy. First-line and second-line or beyond were accounted for 32.3 and 67.8%. A flow chart of the study is shown in Figure 4.

### Association Between Pretreatment NLR and Clinical Outcomes

**Figure 5:**
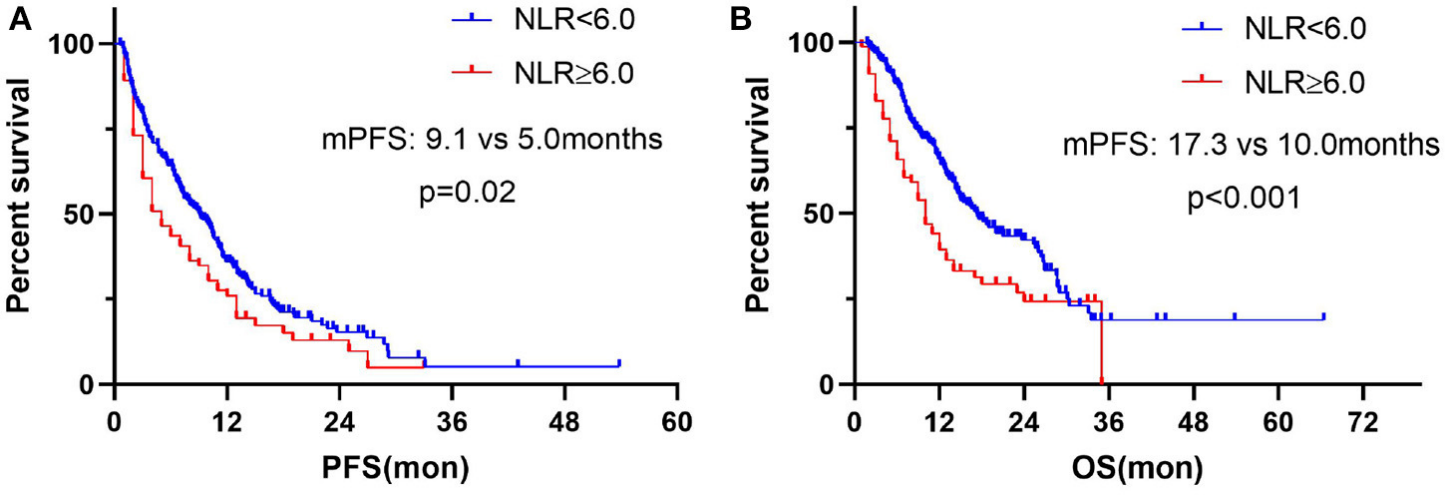
Correlation analysis between pretreatment NLR and clinical outcomes: **(A)** Association between pretreatment NLR and PFS. **(B)** Association between pretreatment NLR and OS.

**Figure 6:**
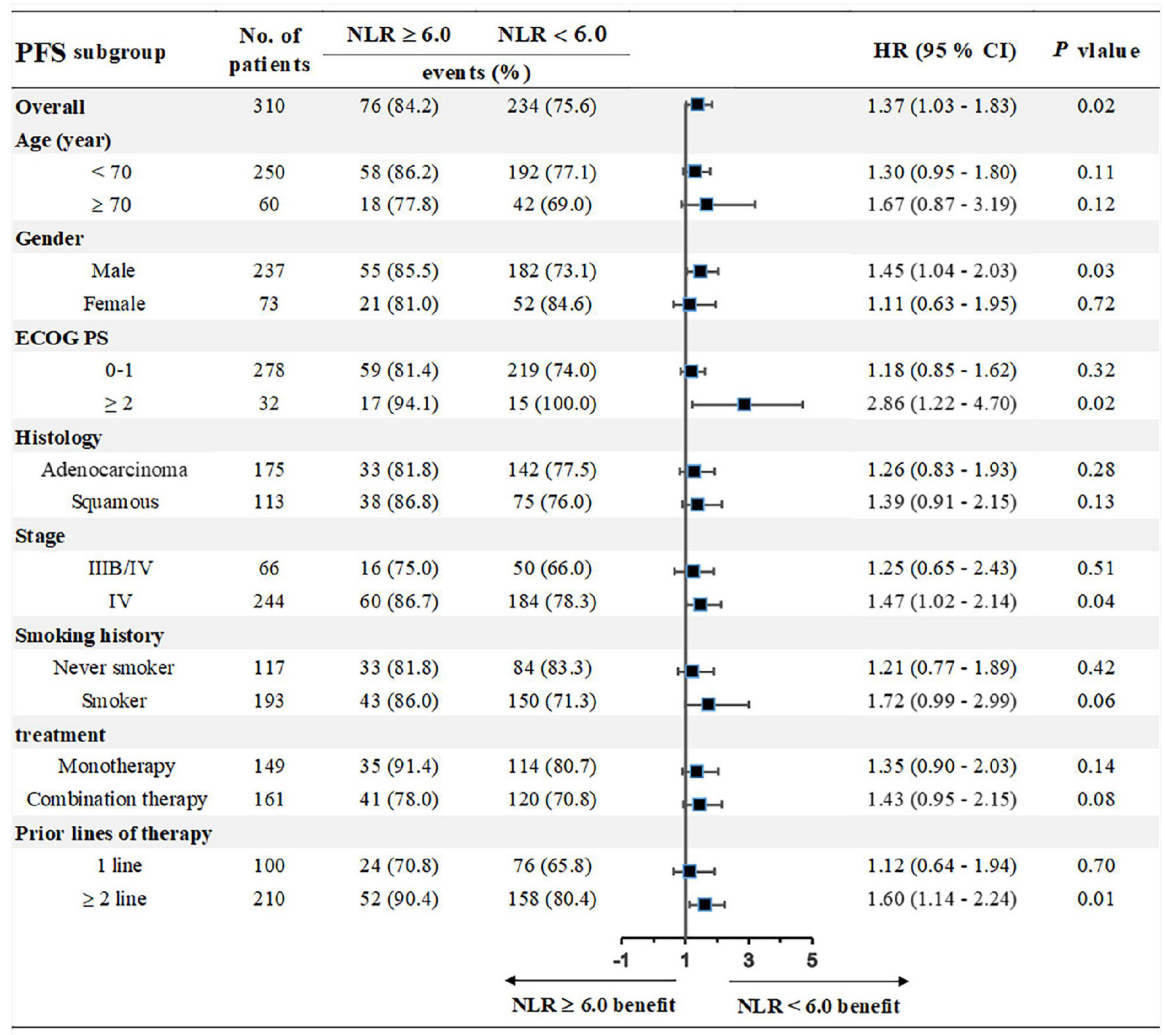
Subgroup analyses of the associations between pretreatment NLR and PFS.

**Figure 7:**
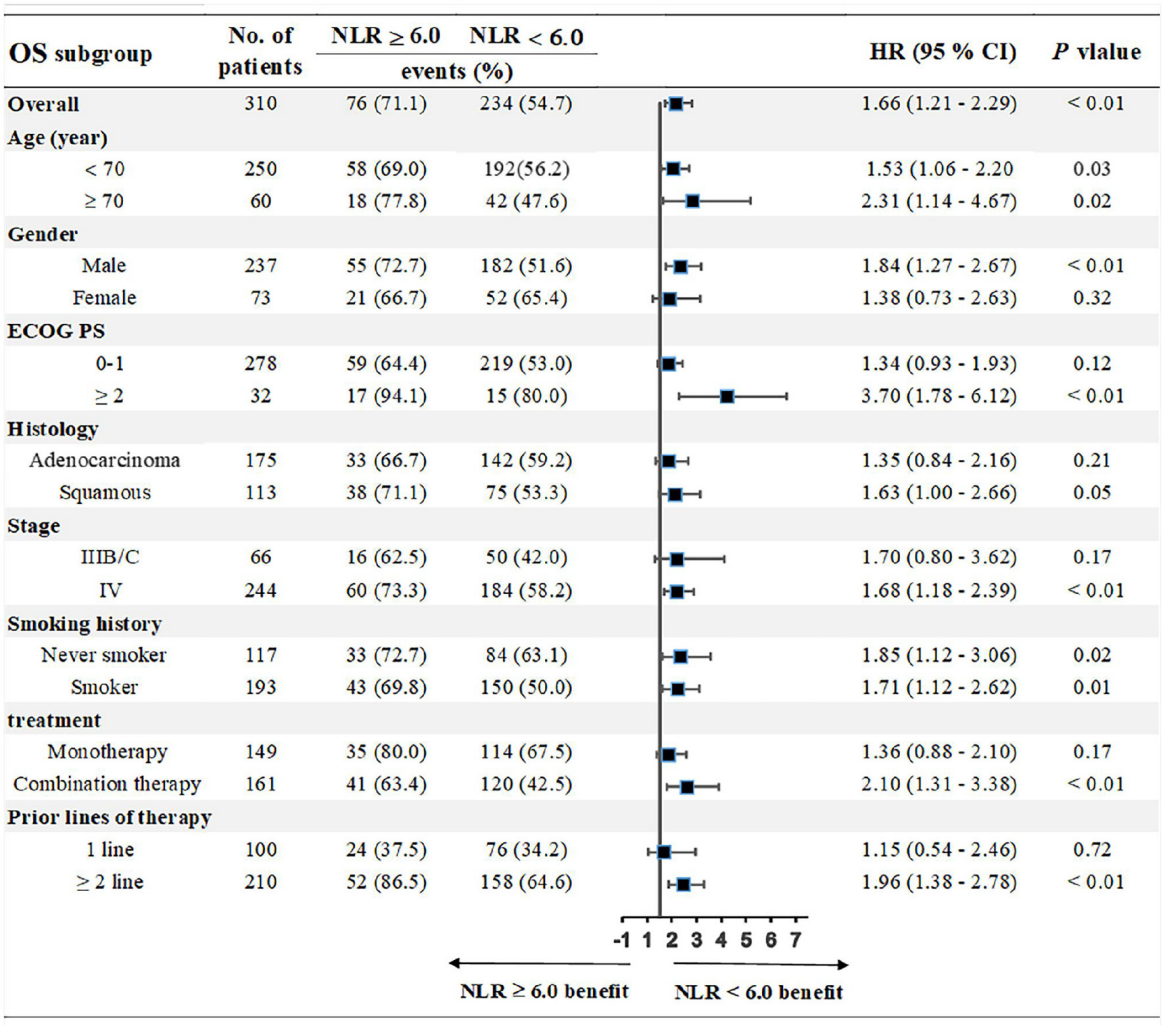
Subgroup analyses of the associations between pretreatment NLR and OS.

We chose the third quartile baseline NLR (6.0) as the cut-off value to further analyze the clinical outcomes. The results showed that patients with pretreatment NLR ≥ 6.0 had shorter PFS (median: 5.0 vs. 9.1 months; HR = 1.39, 95% CI: 1.01–1.91; *P* = 0.02) and OS (median: 10.0 vs. 17.3 months; HR = 1.71, 95% CI: 1.18–2.46; *P* < 0.001) than those with NLR < 6.0 (Figure 5). Subgroup analyses still showed that pretreatment NLR ≥ 6.0 was a risk factor for both PFS and OS in almost all of the subgroups, when stratified by age, gender, ECOG PS, histology, stage, smoking history, treatment, and prior lines of therapy (Figures 6, 7).

### Association Between Dynamics of NLR and Clinical Outcomes

**Figure 8:**
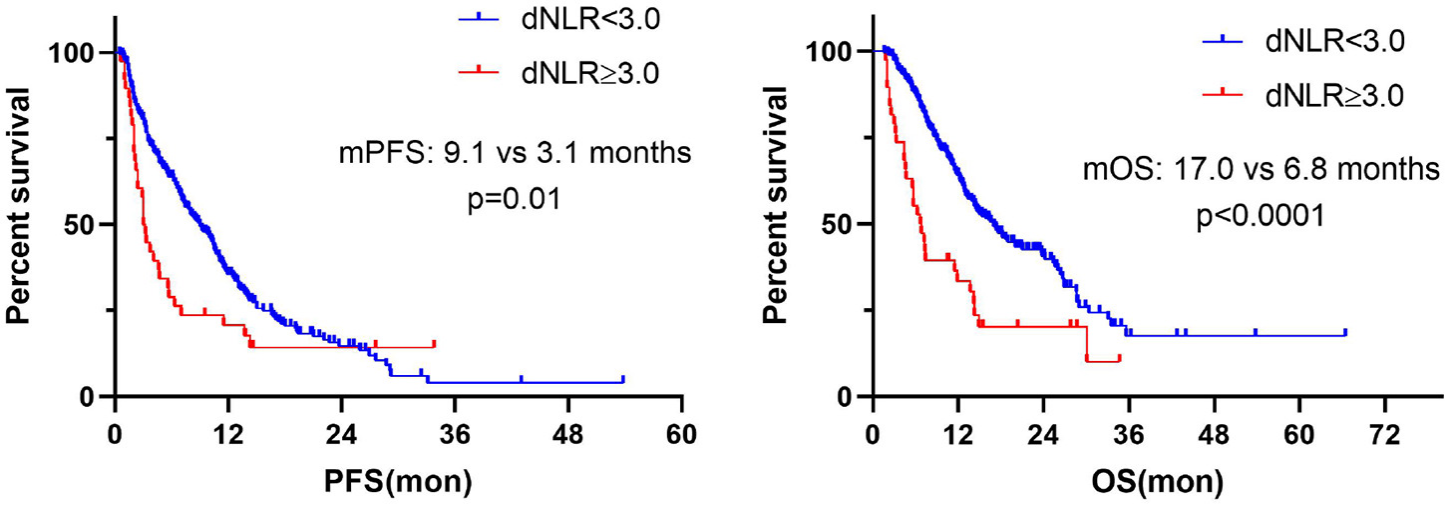
Dynamic change in NLR associated with PFS and OS.

Further, we calculated the dynamics of NLR (dNLR) after 6 weeks from baseline and divided the population into dNLR < 3.0 group and dNLR ≥ 3.0 group by median value of dNLR. The result showed that dNLR increased ≥3.0 after 6 weeks from baseline and was significantly associated with worse PFS (median: 3.1 vs. 9.1 months; *P* = 0.01) and OS (median: 6.8 vs. 17.0 months; *P* < 0.0001) (Figure 8).

## Discussion

In the present study, we first explored the association between pretreatment NLR and clinical outcomes in NSCLC patients receiving immunotherapy in previously published studies, and found that high pretreatment NLR values corresponded to poorer PFS and OS. We further validated the results in our retrospective cohort. Taken together, our results suggest that NLR may be a potential peripheral blood biomarker and an effective tool to stratify patients who are likely to benefit from ICI therapy.

Inflammation plays a key role in tumor development, affecting the survival of cancer patients (47–49). The utility of NLR lies in its ability to reflect the degree of inflammation in a patient's body (7, 14, 50), and a series of studies have confirmed its relationship with tumor prognosis (7, 51–54). The relationship between tumor and inflammation has attracted wide attention since as early as the nineteenth century, when Rudolf Virchow discovered the presence of leukocytes in tumor tissues, and the potential relationship between tumor and inflammation was first proposed (55). Epidemiological studies have demonstrated that ~25% of cancer cases can be attributed to infection and chronic inflammation (56). In addition, inflammation can promote tumorigenesis by secreting growth factors or cytokines or inducing gene mutations (57, 58).

The occurrence and development of malignant tumors are affected by the tumor microenvironment (TME) and the immune system (59, 60). Growing evidence suggests that both neutrophils and lymphocytes, components of the immune system, are involved in tumor progression and prognosis. The presence of neutrophils in peripheral blood indicates inflammation, and lymphocytes in peripheral blood are important indicators of the immune system, the latter of which plays an indispensable role in the pathogenesis of lung cancer (59).

As a critical component of the inflammatory response, neutrophils not only target tumor cells but also indirectly act on the TME, driving or promoting tumor development (61). On one hand, neutrophils secrete tumor growth factors, cytokines, and chemokines, including TGF-beta, VEGF, IL-6, IL-8, IL-12, and matrix metalloproteinase, which can promote angiogenesis (15, 62). On the other hand, tumor cells release granulocyte colony-stimulating factor (G-CSF), which can increase the number of neutrophils. Thus, a mutually reinforcing relationship exists between neutrophils and tumor cells (63). A recent study shows that neutrophils in NSCLC act to inhibit anti-tumor immune responses by inhibiting the cytotoxic activity of immune cells, particularly activated T cells (64, 65).

Lymphocytes are a significant component of human cellular immunity and are involved in anti-tumor immune responses. In particular, T lymphocytes are crucial to the recognition and killing of tumor cells, thereby inhibiting tumor cell proliferation and metastasis (66, 67). Reduction in lymphocyte count reduces the anti-tumor effect of the immune system, resulting in accelerated tumor occurrence and development (67). Lymphocyte decrease also weakens the effectiveness of ICIs, which mainly unleashes the inhibitory signal function of T lymphocytes. Studies have demonstrated that increased lymphocyte infiltration in tumor and TME is associated with a better response to immunotherapy and prognosis in solid tumor patients (68). TME is an important factor in cancer progression, immune escape, invasion, and distant metastasis (69).

Given the roles of neutrophils and lymphocytes in tumor growth, changes in NLR can reflect the body's anti-tumor status (66). Increase in NLR suggests increase in the absolute number of neutrophils and/or decrease in the absolute number of lymphocytes and, thus, decrease in the anti-tumor effect of the immune system. These changes are associated with a poor response to immunotherapy in cancer patients. Conversely, decrease in NLR may indicate improved anti-tumor effect and good response to immunotherapy. Emerging evidence suggests that an increased NLR is a reliable hematologic indicator of poor prognosis in NSCLC (7, 70).

Although the cut-off value of NLR in our study was different from previous studies, we found that pretreatment and dynamic change in NLR was significantly associated with prognosis of patients receiving ICI treatment. Further studies in large scale are needed to confirm the predictive value of pretreatment NLR in advanced NSCLC patients treated with ICIs.

In conclusion, the current study demonstrates that high pretreatment and increased NLR after immunotherapy are associated with poor outcomes of advanced NSCLC patients with ICI treatment. Our results suggest that pretreatment NLR ≥ 6.0 and NLR increase ≥3.0 after ICI treatment are associated with significant poor PFS and OS. NLR is a promising biomarker of the prognosis of advanced NSCLC patients receiving ICIs, which warrants further prospective studies.

## Data Availability Statement

The datasets generated for this study are available on request to the corresponding author.

## Ethics Statement

The study protocol was approved by the Ethics Committee of Chinese PLA general hospital. The patients/participants provided their written informed consent to participate in this study.

## Author Contributions

JW and SJ conceived the idea of this article. XY, QS, GW, and RC completed the work of acquisition of data. YL and ZZ shared the task of analysis, interpretation of data, and manuscript writing. All authors participated in discussing and revising the manuscript.

## Conflict of Interest

GW is an employee of 3D Medicines. The remaining authors declare that the research was conducted in the absence of any commercial or financial relationships that could be construed as a potential conflict of interest.
